# Phytosulfokine-α Controls Hypocotyl Length and Cell Expansion in *Arabidopsis thaliana* through Phytosulfokine Receptor 1

**DOI:** 10.1371/journal.pone.0021054

**Published:** 2011-06-16

**Authors:** Nils Stührwohldt, Renate I. Dahlke, Bianka Steffens, Amanda Johnson, Margret Sauter

**Affiliations:** Entwicklungsbiologie und Physiologie der Pflanzen, Universität Kiel, Kiel, Germany; Ecole Normale Superieure, France

## Abstract

The disulfated peptide growth factor phytosulfokine-α (PSK-α) is perceived by LRR receptor kinases. In this study, a role for PSK signaling through PSK receptor PSKR1 in *Arabidopsis thaliana* hypocotyl cell elongation is established. Hypocotyls of etiolated *pskr1-2* and *pskr1-3* seedlings, but not of *pskr2-1* seedlings were shorter than wt due to reduced cell elongation. Treatment with PSK-α did not promote hypocotyl growth indicating that PSK levels were saturating. Tyrosylprotein sulfotransferase (TPST) is responsible for sulfation and hence activation of the PSK precursor. The *tpst-1* mutant displayed shorter hypocotyls with shorter cells than wt. Treatment of *tpst-1* seedlings with PSK-α partially restored elongation growth in a dose-dependent manner. Hypocotyl elongation was significantly enhanced in *tpst-1* seedlings at nanomolar PSK-α concentrations. Cell expansion was studied in hypocotyl protoplasts. WT and *pskr2-1* protoplasts expanded in the presence of PSK-α in a dose-dependent manner. By contrast, *pskr1-2* and *pskr1-3* protoplasts were unresponsive to PSK-α. Protoplast swelling in response to PSK-α was unaffected by ortho-vanadate, which inhibits the plasma membrane H^+^-ATPase. In maize (*Zea mays* L.), coleoptile protoplast expansion was similarly induced by PSK-α in a dose-dependent manner and was dependent on the presence of K^+^ in the media. In conclusion, PSK-α signaling of hypocotyl elongation and protoplast expansion occurs through PSKR1 and likely involves K^+^ uptake, but does not require extracellular acidification by the plasma membrane H^+^-ATPase.

## Introduction

Phytosulfokine-α (PSK-α) is a disulfated pentapeptide of the sequence Tyr(SO_3_H)-Ile-Tyr(SO_3_H)-Thr-Gln [1], [2]. It is encoded as a preproprotein by five genes in Arabidopsis (*Arabidopsis thaliana*) [3], [4]. Tyrosylprotein sulfotransferase (TPST) was shown to catalyze sulfation of the PSK precursor protein which is required for peptide activity [5]. The active PSK peptide is perceived by plasma membrane-localized leucine-rich repeat (LRR) receptor kinases [6]. In *Arabidopsis thaliana*, two genes encode for PSK receptors, *PSKR1* and *PSKR2*. PSK-α acts as an autocrine growth factor that promotes proliferation of suspension-cultured cells kept at low density [7] and of calli [8]. In Arabidopsis seedlings, PSK-α was shown to regulate root growth [9], [10]. Analysis of *pskr1-3* (previously termed *pskr1-T*) and *pskr2-1* T-DNA insertion lines indicated that root elongation was predominately controlled through PSKR1. PSKR1 signaling altered root growth mainly by increasing cell size [10].

Arabidopsis hypocotyl elongation is under the control of several plant hormones [11]. In etiolated seedlings, positive regulation is exerted by gibberellin and brassinosteroid signaling, while ethylene strongly inhibits hypocotyl elongation. Auxin appears to play a dual role by promoting both hypocotyl elongation and ethylene-mediated inhibition of hypocotyl elongation. A role for PSK signaling in the regulation of hypocotyl growth has not previously been described.

Growth of cells is an irreversible increase in cell volume and is achieved in plants by an increase in cell wall extensibility, by the cells' osmotic potential that manifests itself as turgor pressure, and through water uptake driven by an increase in turgor pressure. The main solutes involved in osmoregulation are K^+^, sucrose, and accompanying anions such as malate and chloride. K^+^ has a high membrane permeability due to the presence of numerous K^+^ channels and transporters. In Arabidopsis, the shaker-like K^+^ channel KAT1 and the K^+^ transporter KT2/KUP2 have been implicated in cell elongation in the hypocotyl [12], [13]. The phytotoxin fusicoccin (FC) causes plant cells to excrete protons by activating the plasma membrane (PM) H^+^-ATPase and to thereby promote cell growth and protoplast expansion [14]. FC stabilizes binding of a 14-3-3 protein to the C-terminus thus locking the H^+^-ATPase in a permanently activated state [15]. H^+^ acts as counterion of K^+^. By stimulation of net K^+^ uptake and inhibition of K^+^ outward rectifier channels, FC promotes a high turgor pressure and hence cell expansion [16], [17]. Ortho-vanadate acts as an inhibitor of the plant PM H^+^-ATPase and is commonly used to study proton translocating ATPase activity [18], [19]. Vanadate sensitivity of the PM proton pump results from the formation of a covalently bound phosphate intermediate during the reaction cycle [20].

In this study we identified a promotive effect of PSK-α signaling through PSKR1 on hypocotyl cell length and consequently on hypocotyl length in Arabidopsis. Analysis of protoplasts from the hypocotyl indicated that PSK signaling controls osmotically-driven cell expansion indicating that PSK-α likely acts as an osmoregulator.

## Results

### PSK signaling through PSKR1 controls hypocotyl length in Arabidopsis

**Figure 1:**
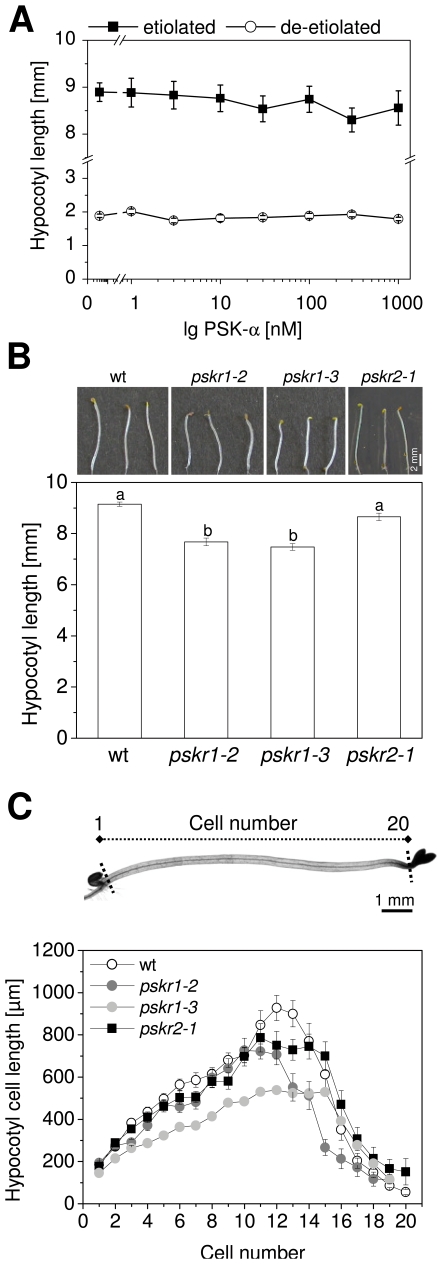
*pskr1-2* and *pskr1-3* seedlings have shorter hypocotyls due to shorter cells. (A) Arabidopsis seedlings were grown on media containing PSK-α at concentrations between 1 nM and 1 µM or without PSK-α for 5 days at long-day conditions or in the dark. Results are averages (± SE) of a minimum of >35 hypocotyls analyzed per treatment in 3 independent experiments. No PSK-α-dependent growth response was observed (*P*<0.001, ANOVA, Tukey test). (B) Hypocotyls of representative wt, *pskr1-2*, *pskr1-3*, and *pskr2-1* seedlings grown for 5 days in the dark. Average (± SE) hypocotyl lengths determined in 3 independent biological experiments. Letters indicate significantly different values (n≥120, *P*<0.001, ANOVA, Tukey test). (C) Hypocotyl cell lengths of wt, *pskr1-2*, *pskr1-3*, and *pskr2-1* seedlings were analyzed and plotted against the cell position. On top, cell numbering of a 5-day-old etiolated wt seedling is indicated from base to top. Cell lengths are averages (± SE) of 20 hypocotyls analyzed per genotype in 2 independent experiments. Cell numbers did not differ significantly between genotypes (*P*<0.05, ANOVA, Tukey test; see also Table S1 in Supporting Information S1).

PSK-α was previously shown to control cell elongation in roots of Arabidopsis [10]. To analyze a possible function of PSK-α in shoot growth, Arabidopsis seedlings were grown on media lacking PSK-α, or on media supplemented with PSK-α at concentrations between 1 nM and 1 µM. Hypocotyl lengths were measured after 5 days of treatment. No effect of PSK-α was observed on hypocotyl elongation in dark-grown seedlings or in de-etiolated seedlings (Figure 1A). We next employed the Arabidopsis T-DNA insertion lines *pskr1-2*, *pskr1-3* and *pskr2-1* that were shown to be deficient in transcripts of the respective PSK receptor genes *PSKR1* and *PSKR2* [8], [10]. Hypocotyl elongation of etiolated *pskr1-2*, *pskr1-3* and *pskr2-1* seedlings was analyzed after 5, 10, 15, 20 and 25 days and was compared to hypocotyl growth of wild type seedlings (Supporting Information S1). Hypocotyls of *pskr1-2* and *pskr1-3* seedlings were shorter after 5 days of growth and remained shorter than hypocotyls of wild type whereas hypocotyls of *pskr2-1* seedlings reached wt length (Figure 1B, Supporting Information S1). These results indicated that PSK signaling specifically through PSKR1 has a positive impact on seedling shoot elongation in the dark.

### PSK signaling through PSKR1 promotes cell elongation

Arabidopsis hypocotyls possess about 20 epidermal cells from the base to the apical hook with epidermal cells reaching a length of up to 1 mm in the dark [21]. To find out if PSKR1 activity was required for cell elongation or for cell division, hypocotyl cell lengths and numbers were determined in 5-day-old etiolated wt, *pskr1-2*, *pskr1-3*, and *pskr2-1* seedlings (Figure 1C). The average number of cells was between 19 and 20 in the various genotypes. Differences in cell number were not statistically significant (Supporting Information S1, *P*<0.05, ANOVA, Tukey test). Average hypocotyl cell lengths were determined in 5-day-old seedlings at each cell position starting at the base of the hypocotyl. The longest cells were found at cell positions 11 to 13 in wt and reached a maximum length of about 930 µm. Cells 3 to 14 of *pskr1-3* hypocotyls were significantly shorter than the corresponding cells of wt (Supporting Information S1). In *pskr1-2* seedlings, the cell growth reduction was somewhat less pronounced than in *pskr1-3*, but still significant at many cell positions (Supporting Information S1). In *pskr2-1* seedlings, one cell at position 13 was significantly shorter than the corresponding wt cell, but all other cells were as large as in wt (Supporting Information S1) resulting in a net wt-like hypocotyl length. These results support the view that PSK signaling through PSKR1 contributes to hypocotyl cell elongation. A minor effect of PSKR1 knock out on cell division activity cannot be completely excluded although statistics do not support this.

### *PSK* and *PSKR* gene expression in the seedling shoot

**Figure 2:**
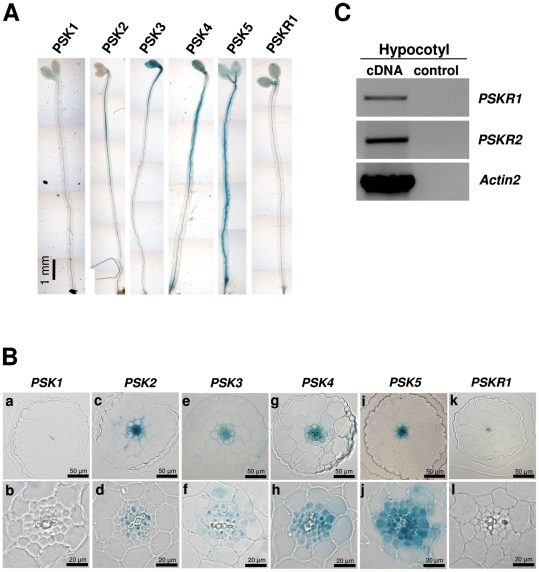
Expression analysis of PSK and PSK receptor genes in seedling shoots. (A) Promoter:GUS analysis of 5-day-old etiolated *PSK1:GUS*, *PSK2:GUS*, *PSK3:GUS*, *PSK4:GUS*, *PSK5:GUS*, and *PSKR1:GUS* seedlings. (B) Cross sections from hypocotyls indicating activity of *PSK2:GUS*, *PSK3:GUS*, *PSK4:GUS*, and *PSK5:GUS* in 5-day-old etiolated seedlings. (C) RT-PCR analysis of *PSKR1* and *PSKR2* expression in hypocotyls of 5-day-old etiolated Arabidopsis seedlings. *Actin2* cDNA was amplified as a control for RNA input. As a control for contamination with genomic DNA, RNA was added to PCR reactions.

Since *pskr1-2* and *pskr1-3* seedlings had shorter hypocotyls than wt, expression of *PSKR1* and of PSK genes was analyzed in the shoot of etiolated Arabidopsis seedlings. To this end, promoter:GUS lines were employed [10]. In hypocotyls, GUS activity was observed for *PSK2:GUS*, *PSK3:GUS*, *PSK4:GUS*, and *PSK5:GUS* (Figure 2A). To look at cell-specific expression of PSK genes, cross sections at the upper third of hypocotyls were made. Strongest GUS staining appeared in the vascular cylinder of *PSK2:GUS*, *PSK3:GUS*, *PSK4:GUS*, and *PSK5:GUS* seedlings (Figure 2B, c–j). Staining was also obvious in parenchyma cells of *PSK2:GUS*, *PSK3:GUS*, and *PSK4:GUS* seedlings (Figure 2B, ceg) and was predominately found in the upper half of the seedling. No GUS activity was observed in *PSK1:GUS* and *PSKR1:GUS* hypocotyls (Figure 2B, abkl). A more sensitive method was therefore chosen to detect *PSKR* transcripts. PCR amplification of reverse transcribed cDNA indicated that both *PSKR1* and *PSKR2* were expressed in the hypocotyl (Figure 2C), an expression that was supported by microarray data as summarized in the genevestigator database (https://www.genevestigator.com/). In summary, expression analysis revealed that PSKR and PSK genes were active in the hypocotyl.

### *tpst-1* seedlings have shorter hypocotyls and respond to PSK-α in a dose-dependent manner

**Figure 3:**
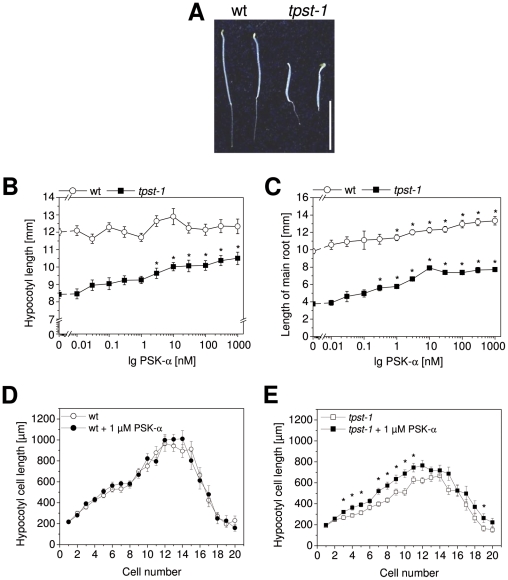
*tpst-1* seedlings have shorter hypocotyls and are responsive to PSK-α. (A) 5-day-old etiolated wt and *tpst-1* seedlings grown without PSK-α. (B) Hypocotyl lengths of etiolated wt and *tpst-1* seedlings treated for 5 days with PSK-α at the concentrations indicated. Asterisks indicate significantly different values to the untreated control. Average (±SE) hypocotyl lengths were determined in 2 independent biological experiments with at least 40 seedlings analyzed per data point (*P*<0.001, 2-sample *t*-test). (C) Root lengths of etiolated wt and *tpst-1* seedlings treated for 5 days with PSK-α at the concentrations indicated. Asterisks indicate significantly different values to the untreated control. Averages (±SE) were determined in 2 independent biological experiments (n≥40; *P*<0.001, 2-sample *t*-test). (D) Hypocotyl cell lengths of 5-day-old etiolated wt seedlings treated with or without 1 µM PSK-α were plotted against the cell position from base to top. Results are averages (±SE) of 20 hypocotyls analyzed in 2 independent experiments. Values are not significantly different (*P*<0.05, 2-sample *t*-test). (E) Hypocotyl cell lengths of etiolated *tpst-1* seedlings that were treated with or without 1 µM PSK-α for 5 days. Results are averages (±SE) of 20 hypocotyls analyzed in 2 independent experiments. Asterisks indicate statistically significant differences (*P*<0.05, 2-sample *t*-test).

Hypocotyls expressed PSK precursor genes and hypocotyl elongation was not promoted by exogenous PSK-α in etiolated wt seedlings. This may have been due to an inability to transport PSK-α from the roots to the hypocotyl or to sites of perception by its receptor. Alternatively, it is conceivable that PSK-α levels might be saturating in the hypocotyl. To test these hypotheses we employed the *tpst-1* mutant which is deficient of active PSK-α due to its inability to carry out tyrosine sulfation of the PSK peptide precursor [5]. Hypocotyls of *tpst-1* seedlings were 30% shorter than those of wt (Figure 3A, B). The number of cells were not different in wt and *tpst-1*; instead cells were shorter (Figure 3D, E). *tpst-1* seedlings responded to PSK-α with significantly enhanced hypocotyl elongation (Figure 3B). The growth promoting effect was observed at 3 nM and higher concentrations. Treatment with PSK-α did however not fully restore wt hypocotyl length. Hypocotyls of *tpst-1* seedlings treated with 1 µM PSK-α were still significantly shorter than those of wt seedlings indicating that additional sulfated growth promoting factor(s) are lacking. At the cell level, PSK-α promoted cell elongation and not cell division (Figure 3E). Dose-dependent growth promotion was also observed for *tpst-1* seedling roots which were significantly longer when exposed to as little as 0.3 nM PSK-α (Figure 3C). Saturation of the growth promoting effect was achieved at 10 nM. As was observed for hypocotyls, wt root length was not fully restored by PSK-α.

### PSK-α promotes protoplast expansion through PSKR1

**Figure 4:**
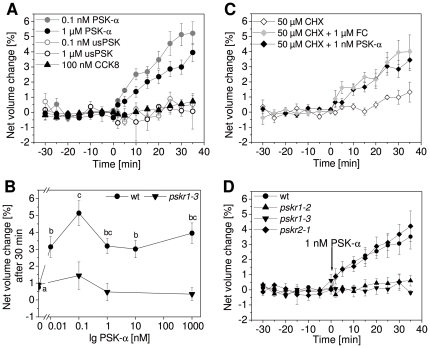
Protoplasts from the Arabidopsis hypocotyl expand in response to PSK-α. Protoplasts were isolated from etiolated hypocotyls and their volume was determined at 5 min intervals. After 30 min, at t = 0 min, effectors were added and protoplast volumes were recorded for another 35 min. (A) Addition of 0.1 nM or 1 µM PSK-α caused a rapid and continuous increase in protoplast volume whereas unsulfated PSK peptide (usPSK) or 100 nM of the sulfated peptide CCK8 did not (n = 3–5, *P*<0.05, 2-sample *t*-test). (B) Dose-response curve of protoplast expansion in wt and *pskr1-3* at PSK-α concentrations between 0.01 nM and 1 µM. The net volume change was determined 30 min after addition of PSK-α. Results are averages (±SE) of 3 to 7 protoplasts analyzed per treatment and genotype. Different letters indicate significantly different values (*P*<0.05, ANOVA, Tukey test). (C) Protoplasts were pre-treated with 50 µM cycloheximide (CHX) for 1 h prior to the addition of 1 µM fusicoccin (FC) or 1 nM PSK-α at t = 0 in addition to CHX. As a control, protoplasts were treated with CHX only. CHX did not inhibit protoplast expansion induced by FC or PSK-α (n = 4–5, *P*<0.05, 2-sample *t*-test). (D) Protoplasts from wt, *pskr1-2*, *pskr1-3*, and *pskr2-1* were treated with 1 nM PSK-α. Protoplasts from wt and *pskr2-1*, but not from *pskr1-2* or *pskr1-3* seedlings expanded. Results are averages (±SE) of 3 to 7 protoplasts analyzed per treatment and genotype. Rates of net volume change of wt and *pskr2-1* are not significantly different (*P*<0.05, 2-sample *t*-test). Expansion of *pskr1-2* or *pskr1-3* protoplasts is significantly different to wt and *pskr2-1* (*P*<0.05, 2-sample *t*-test).

To study a regulatory role of PSK-α in cell expansion through osmotic adjustment, protoplasts were isolated from hypocotyls of etiolated Arabidopsis seedlings. Protoplast volume was calculated from the circumference that was determined for each protoplast analyzed at each time point measured [22]. Protoplast sizes were followed at 5 min intervals over a 30 min period prior to the addition of an effector to make sure that the protoplasts were vital. After effector application, protoplast volumes were determined for another 35 min (Figure 4). Protoplasts isolated from etiolated wt hypocotyls were treated with PSK-α. Addition of 0.1 nM PSK-α resulted in rapid protoplast expansion within few minutes that continued over the 35 min period of observation. Treatment with 1 µM PSK-α also caused protoplast swelling with a slightly weaker response than at 0.1 nM PSK-α. Neither treatment with unsulfated PSK peptide at 0.1 nM or 1 µM nor with 100 nM of the sulfated control peptide CCK8 affected protoplast volume indicating that the response was specific for PSK-α (Figure 4A). To resolve the dose-dependent swelling response, intermediary PSK-α concentrations and a lower concentration were applied (Figure 4B). Significant protoplast expansion was induced by as low as 0.01 nM PSK-α and was elevated at all higher concentrations with a maximum response at 0.1 nM PSK-α. Due to the short lag phase of the swelling response of less than 5 min (Figure 4A) we hypothesized that swelling occurred independent of protein synthesis. To test this hypothesis, protoplasts were pre-treated with 50 µM cycloheximide to inhibit protein synthesis (Figure 4C). Treatment with cycloheximide affected neither lag phase nor degree of protoplast swelling indicating that protoplast expansion in response to PSK-α was mediated through post-translational events.

We next analyzed expansion of hypocotyl protoplasts from the PSK receptor mutants *pskr1-2*, *pskr1-3*, and *pskr2-1*. Knock out of the PSKR2 receptor in *pskr2-1* did not affect the responsiveness of protoplasts yielding a wild type response to treatment with 1 nM PSK-α (Figure 4D). By contrast, *PSKR1* deficient protoplasts that were isolated from hypocotyls of the allelic lines *pskr1-2* and *pskr1-3* were not responsive to 1 nM PSK-α (Figure 4D). A dose response analysis established that *pskr1-3* protoplasts had lost their responsiveness to PSK-α supplied at concentrations as high as 1 µM (Figure 4B). This result indicated that PSK-α induced protoplast expansion was mediated through PSKR1.

### PSK-α induced protoplast expansion is insensitive to ortho-vanadate

**Figure 5:**
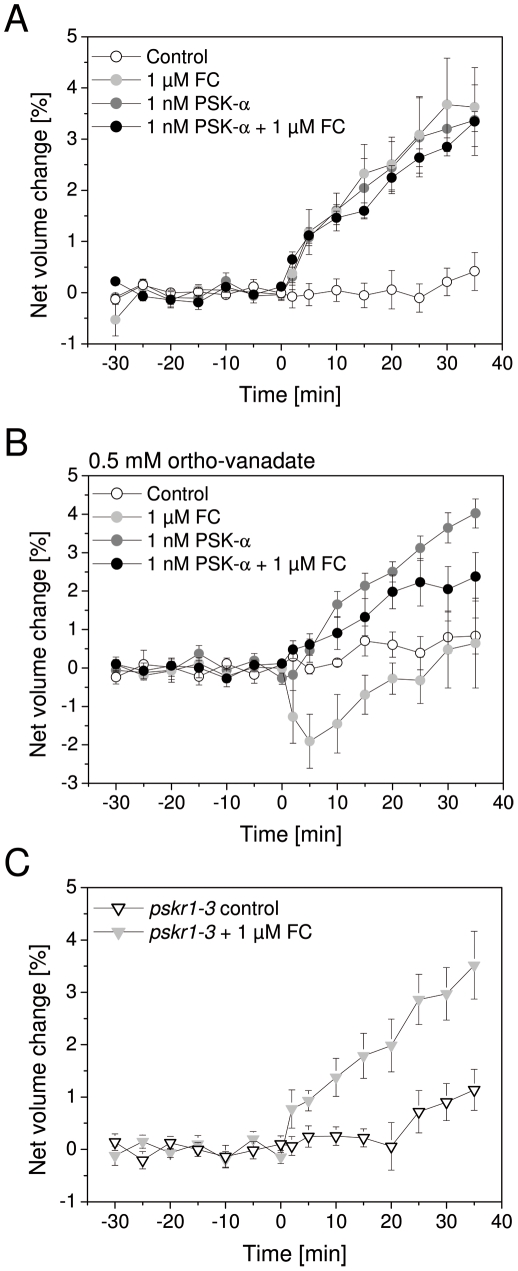
PSK-α induced protoplast swelling is not inhibited by ortho-vanadate. (A) Protoplasts from wt hypocotyls were left untreated (control), or were treated with 1 nM PSK-α, 1 µM FC, or 1 nM PSK-α+1 µM FC at t = 0. Results are averages (±SE) from 4 to 8 protoplasts analyzed per genotype or treatment. Protoplast expansion was not significantly different between effector treatments (*P*<0.05, 2-sample *t*-test). (B) WT protoplasts were pre-treated with 0.5 mM ortho-vanadate for 1 h. At t = 0 min 1 nM PSK-α, 1 µM FC, or 1 nM PSK-α+1 µM FC were added, or protoplasts remained untreated (control). Results are averages (±SE) of 3 or 5 protoplasts analyzed per treatment and genotype. (C) Protoplasts from *pskr1-3* hypocotyls were treated with or without 1 µM FC at t = 0. Protoplasts showed a wt swelling response (see A; *P*<0.05, 2-sample *t*-test).

In order to analyze if proton extrusion was required for PSK-α induced protoplast expansion, the effectors fusicoccin (FC) and ortho-vanadate were used. FC activates the plasma membrane (PM) H^+^-ATPase. WT hypocotyl protoplasts that were treated with 1 µM FC expanded rapidly (Figure 5A). This response was unaffected by cycloheximide treatment as was expected from the known mode of action of FC (Figure 4C). *pskr1-3* protoplasts that were treated with 1 µM FC expanded with a similar kinetic as observed for wt indicating that the capacity for osmoregulation through enhanced proton pump activity was not impaired in *pskr1-3* (Figure 5C). This result is compatible with the view that PSKR1 acts upstream or independent of the PM H^+^-ATPase. We next analyzed protoplast expansion in the presence of ortho-vanadate, an inhibitor of P-type ATPases including the PM H^+^-ATPase [20], [23]. WT protoplasts were preincubated with 0.5 mM ortho-vanadate for 1 h and subsequently treated with or without 1 nM PSK-α. In the presence of ortho-vanadate alone, protoplasts did not change in volume (Figure 5B). When treated with ortho-vanadate and, in addition, with 1 nM PSK-α, protoplasts expanded with a similar kinetic and to a comparable degree as with 1 nM PSK-α in the absence of ortho-vanadate (Figure 5A, B). As a control for ortho-vanadate activity, protoplasts that were pre-treated with 0.5 mM ortho-vanadate for 1 h were subsequently exposed to 1 µM FC. Whereas in the absence of ortho-vanadate, FC induced rapid protoplast swelling, no swelling was observed in the presence of ortho-vanadate (Figure 5B). Instead, protoplasts shrank transiently after FC application in the presence of ortho-vanadate but recovered within 20 min to near control volume. Finally, we treated protoplasts with 1 µM FC and 1 nM PSK-α at the same time. In the absence of ortho-vanadate, protoplasts expanded similarly as protoplasts that were treated with either FC and PSK-α alone possibly indicating that the swelling response achieved with either effector was saturated (Figure 5A). When protoplasts were preincubated with 0.5 mM ortho-vanadate and subsequently exposed to both, FC and PSK-α, protoplasts displayed an intermediary response to that observed with either effector alone (Figure 5B). In other words, PSK-induced protoplast swelling was partially reduced when FC was applied at the same time; and the inhibitory effect of FC was partially reversed with PSK-α.

**Figure 6:**
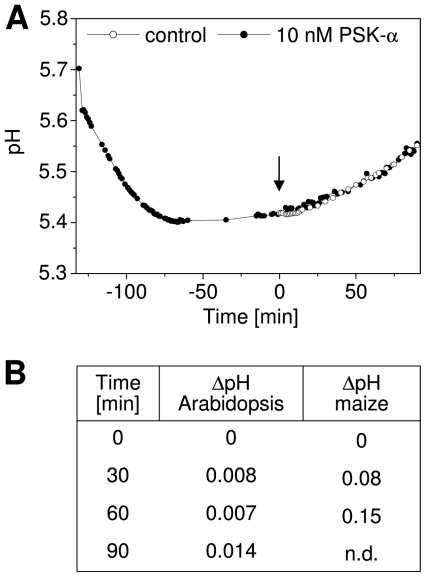
PSK-α does not cause acidification. (A) pH changes in media containing Arabidopsis hypocotyls. At t = 0 hypocotyls were treated with 10 nM PSK-α or remained untreated (control). After hypocotyls were added, the pH of the buffer dropped from pH 5.7 to pH 5.4 within 130 min due to the pH equilibration between tissue and buffer. Subsequently, PSK-α was applied, or hypocotyls remained untreated (arrow). (B) The ΔpH between control and PSK treated hypocotyls was determined after 0, 30, 60, and 90 min. Results are averages of 5 (controls) or 6 (10 nM PSK-α) experiments with 60 hypocotyls each. The increase in pH was not significant at any time point (*P*<0.001, 2-sample *t*-test). In addition, pH changes were determined in media containing 8 coleoptiles from etiolated maize seedlings (n = 3; n.d. - not determined).

Since protoplast expansion appeared to be independent of proton extrusion, we next studied pH changes in hypocotyls that were isolated from seedlings grown in dim light. If PSK-α promoted proton extrusion, the apoplast would be acidified and this acidification can be measured in the incubation medium [24], [25]. During the equilibrium phase of the hypocotyls to the media, the pH in the medium dropped until the acid equilibrium was reached. Addition of 10 nM PSK-α did not result in further acidification. Rather a slight alkalinization was observed as compared to the untreated control (Figure 6A, B). As described below, protoplasts from maize coleoptiles expand in response to PSK-α as do Arabidopsis protoplasts. When maize coleoptiles were treated with 10 nM PSK-α, the medium did also not acidify as was observed for Arabidopsis hypocotyls; rather the pH slightly increased within 1 h. Our results thus indicate that PSK driven cell and protoplast expansion are not accompanied by apoplast or media acidification.

### PSK-α mediated protoplast expansion is dependent on exogenous K^+^

**Figure 7:**
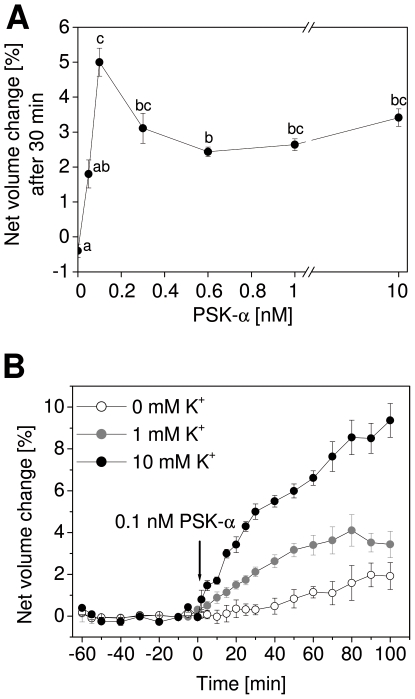
Maize protoplasts expand in a K^+^-dependent manner. (A) Protoplasts were isolated from maize coleoptiles and treated with PSK-α at concentrations between 0.05 nM and 10 nM. The dose-response curve was highly similar to that observed with Arabidopsis hypocotyl protoplasts with a maximal response at 0.1 nM PSK-α. Results are averages (±SE) from 3 to 5 protoplasts analyzed per treatment. Letters indicate significantly different values (*P*<0.001, ANOVA, Tukey test). (B) Maize coleoptile protoplasts were incubated in media containing varying concentrations of K^+^. After addition of 0.1 nM PSK-α at t = 0 min, protoplast swelling was recorded for another 100 min. Maximal protoplast expansion was observed at 10 mM K^+^ whereas a weaker response was observed at 1 mM K^+^. Without K^+^ in the media, protoplasts did not expand (*P*<0.001, ANOVA, Tukey test).

When plant cells expand, K^+^ very often acts as osmotically active ion that is taken up from the surrounding apoplast. The solution in which protoplasts were incubated contained 10 mM K^+^. To investigate a possible role of potassium in PSK-α-dependent expansion, protoplasts were incubated in media containing less or no K^+^. However, Arabidopsis protoplasts were not stable in solution lacking K^+^. We therefore used protoplasts isolated from maize coleoptiles for these experiments. Coleoptiles are known to grow exclusively through cell expansion. A dose-response analysis performed on maize protoplasts showed swelling in response to PSK-α at concentrations as low as 0.05 nM and up to 10 nM (Figure 7A). Higher concentrations were not tested. As was observed for Arabidopsis protoplasts, expansion occurred with a very short lag (Figure 7B), and was maximal at 0.1 nM PSK-α (Figures 4B and 7A). Treatment with 0.1 nM unsulfated control peptide did not induce maize coleoptile protoplast swelling (data not shown). In order to test a requirement of K^+^, maize protoplasts were subsequently incubated in media containing no K^+^, 1 mM K^+^, or 10 mM K^+^ (Figure 7B). At 1 mM K^+^, PSK-α induced protoplast swelling was reduced by about 60% as compared to the expansion observed with 10 mM K^+^. In the absence of K^+^, PSK-α did not induce significant protoplast swelling. Hence, PSK-α induced water uptake into protoplasts was dependent on the availability of K^+^ in the media in a dose-dependent manner.

## Discussion

PSK-α acts as an autocrine growth factor that is produced in the secretory system, released into the apoplast, and perceived by plasma membrane-localized LRR receptor kinases. In Arabidopsis, two PSK receptor proteins, PSKR1 and PSKR2, perceive PSK-α [6], [9]. Several physiological responses to PSK-α have been observed, one of which is growth regulation. Suspension cells of Arabidopsis that were treated with PSK-α developed larger calli. Similarly, transgenic Arabidopsis cells overexpressing the PSK-α preproprotein gene *PSK4* developed larger calli as compared to wt, and transgenic cells lacking detectable levels of PSKR1 protein developed smaller calli [9]. *In planta* studies in Arabidopsis revealed that seedling roots were shorter in *pskr1-2* and *pskr1-3* than in wt indicating that root elongation was dependent on PSK signaling through PSKR1 [9], [10]. A minor effect of PSKR2 on root elongation was also observed.

The current study showed that not only roots but also hypocotyls were shorter in etiolated *pskr1-2* and *pskr1-3* seedlings. Knock out of PSKR2 did not significantly affect hypocotyl length indicating that the two receptor proteins have distinct functions. Exogenous supply of PSK-α did not induce growth in etiolated or de-etiolated seedlings. The short hypocotyl phenotype of the *pskr1* knock out lines and the inefficiency of exogenous PSK-α to promote growth led us to hypothesize that hypocotyl elongation was controlled by PSK signaling and that PSK-α was present at saturating amounts in wt hypocotyls. The PSK preproprotein genes *PSK2*, *PSK3*, *PSK4*, and *PSK5* were found to be active in the hypocotyl as revealed in the respective promoter:GUS lines indicating that PSK-α was synthesized in this organ. Promoters of *PSK2*, *PSK3*, and *PSK4* were particularly active in the upper half of the hypocotyl where the strongest effect on cell elongation was observed [21].

To test the hypothesis that wt hypocotyls produce saturating levels of PSK-α for growth promotion, we employed the *tpst-1* mutant. TPST catalyzes the transfer of sulfate to the two tyrosyl side chains of PSK [5]. The *tpst-1* knock out line hence lacks the ability to synthesize functional PSK-α. *tpst-1* seedlings displayed shorter hypocotyls, a phenotype that was partially restored by the addition of PSK-α. This result indicated that PSK-α was delivered to *tpst-1* hypocotyls and was perceived there. However, *tpst-1* hypocotyls remained shorter than wt hypocotyls even when supplemented with high PSK-α concentrations which may still indicate limited accessibility of exogenously supplied PSK-α. It is likewise conceivable that aside from PSK-α one or more additional sulfated peptides contribute to regulation of hypocotyl growth. Similar to *pskr1* seedlings, *tpst-1* seedlings had a wt number of cells which were shorter than in wt. Taken these data together it can be concluded that PSK signaling controls hypocotyl cell elongation. *tpst-1* seedlings also had shorter roots as was reported previously [5], [26]. The wt root length was restored by the addition of PSK-α and of another sulfated peptide PSY1, each supplied at 100 nM [27]. It is conceivable that PSY1 also participates in the regulation of hypocotyl growth. A dose-response analysis on *tpst-1* seedlings showed that root elongation was significantly promoted by as little as 0.3 nM PSK-α indicative of high affinity ligand binding and was saturated at 10 nM PSK-α. In wt seedlings, root growth was significantly enhanced at ≥10 nM PSK-α [10]. *tpst-1* seedlings thus appeared to be more responsive to PSK-α than wt.

*PSKR1* was expressed at low levels in the hypocotyl. While no *PSKR1:GUS* signal was detectable, *PSKR1* transcripts were amplified by RT-PCR. This is in agreement with an earlier report showing overall low *PSKR1* expression in Arabidopsis roots and shoots [9], [10]. Expression of PSK genes was strongest in the central cylinder but low-level expression was also observed in other cell types. The epidermis was discussed as being the growth-limiting cell layer [28]; future studies should clarify if PSK signaling in the epidermis is required for growth promotion.

Reduced root length in *pskr1-2* and *pskr1-3* was largely caused by reduced cell length rather than altered cell production providing evidence that PSK-α controls cell growth [10]. Regulation of cell size through PSKR1 signaling was confirmed in this study which shows that lengths of epidermal cells in the hypocotyl were shorter in *pskr1* and in *tpst-1* knock out lines. Cell expansion requires that water is taken up. Water uptake is driven by a difference in osmotic potential between protoplast and apoplast and requires yielding of the cell wall that surrounds the protoplast. To test the hypothesis that PSK-α signaling positively affects osmoregulation, protoplasts were employed. Protoplasts lack a cell wall and are thus suitable to measure osmotically driven expansion independent of changes in cell wall properties. WT protoplasts expanded rapidly in response to PSK-α, but not when incubated with unsulfated control peptide indicating that the response was specific. An increase in protoplast volume was observed within a short lag phase of 2 to 5 min after application of PSK-α and occurred independent of protein synthesis. As was found for hypocotyl growth, protoplast expansion was mediated through the PSK receptor PSKR1. *pskr1-3*, which lacks detectable *PSKR1* transcript or PSKR1 protein levels [9], [10], was unresponsive to PSK-α over a wide concentration range between 0.01 nM and 1 µM PSK-α. By contrast, protoplasts isolated from Arabidopsis seedlings that lack the second PSK receptor, PSKR2, showed a wt response. In suspension cells from rice, high-affinity binding of PSK-α to plasma membranes with a K_D_ value of 1.4 nM and low-affinity binding with a K_D_ value of 27 nM was described [29]. The binding affinity to Arabidopsis microsomes was determined with a K_D_ of 7.7 nM [9]. A maximal protoplast swelling response was induced at 0.1 nM PSK-α, and root elongation was promoted at 0.3 nM PSK-α which is one order of magnitude lower than the dissociation constant measured in Arabidopsis. This may indicate that only few receptor proteins need to be activated in order to induce a response. On the other hand, *in vivo* conditions of receptor-ligand binding may differ from those provided in binding studies on isolated membrane vesicles. Brassinosteroids are effective at similarly low concentrations as PSK-α [30]. Incidentally, the brassinosteroid receptor BRI1 is an LRR receptor kinase that shares high sequence and structural similarity with PSK receptors. For BRI1 and for the carrot PSK receptor DcPSKR1 the respective ligands were shown to bind to the single island domain located in the extracellular LRR region which appears to be particularly suited for high-affinity ligand binding [31], [32].

Rapid protoplast swelling can depend on different mechanisms. Auxin-induced growth and protoplasts expansion are dependent on the activation of the PM H^+^-ATPase [22] and on K^+^ channels [33]. The PM H^+^-ATPase is constitutively activated by FC [15]. Protoplasts isolated from *pskr1-3* hypocotyls were not impaired in FC-induced swelling indicating that PSKR1 was not required for this response possibly because it acts upstream or independent of the PM H^+^-ATPase. Inhibition of P-type ATPases such as the PM H^+^-ATPase by ortho-vanadate did not inhibit protoplast swelling in the presence of PSK-α indicating that PSKR1 mediated protoplast expansion was independent of PM H^+^-ATPase activation. Neither did *in vivo* treatment of Arabidopsis hypocotyls or maize coleoptiles with PSK-α result in medium acidification. For a comparison, treatment of maize coleoptiles with indole-3-acetic acid caused a drop in pH of 0.2 units [25]. A requirement for K^+^ as an osmolyte in PSK-α induced protoplast expansion was supported by the observation that a reduction in K^+^ concentration resulted in reduced swelling of maize protoplasts in a dose-dependent manner. It is possible that PSK-α exerts an effect on K^+^ transport in a direct or indirect manner through as yet unidentified signaling proteins. Both, signaling proteins and targets of PSK signaling are present and likely posttranslationally regulated in PSK responsive protoplasts.

A role for PSK signaling through PSKR1 in hypocotyl growth is supported by the observations that *pskr1* and *tpst-1* seedlings displayed shorter hypocotyls and shorter cells. Most notably, hypocotyl cell elongation of *tpst-1* seedlings was promoted by PSK-α. Furthermore, PSK-α promoted rapid expansion of protoplasts obtained from hypocotyls of wt and *pskr2-1*, but not of protoplasts obtained from hypocotyls of *pskr1-2* or *pskr1-3*. PSK-α induced protoplast swelling was observed in the monocot species maize as well as in the dicot species Arabidopsis indicating that it is a conserved response in higher plants. While a requirement for PM H^+^-ATPase activity was not observed, protoplast expansion induced by PSK-α depended on the presence of K^+^ in the media, indicating that potassium uptake into protoplasts drives water uptake. Regulation of potassium channel activity may thus be a target of PSK signaling. Since the swelling response of protoplasts in response to PSK-α was observed within few minutes and was independent of protein synthesis, regulation of K^+^ or other channel activities likely occur at the protein level.

## Materials and Methods

### Plant material and growth measurements

Experiments with *Arabidopsis thaliana* were performed on ecotype Columbia-0. The *pskr1-2*, *pskr1-3* (previously named *pskr1-T*), and *pskr2-1* insertion lines and P:GUS lines used in this study were described [8]–[10]. The T-DNA is inserted 48 bp downstream of the translational start site in *pskr1-2*, in the kinase domain in *pskr1-3* and in the 11^th^ LRR domain in *pskr2-1*. The *tpst-1* (*At1g08030*) T-DNA insertion line SALK_009847 [5] was obtained from the NASC (Nottingham Arabidopsis Stock Centre, University of Nottingham, Nottingham, UK) and homozygous plants were identified. This insertion line was previously complemented with the wt TPST allele resulting in wt growth indicating that TPST knock out is causal to the growth defect [5]. Arabidopsis seeds were surface-sterilized for 5 min in 70% (v/v) ethanol, washed twice with autoclaved water followed by 30 min in 1 ml 2% (w/v) sodium hypochlorite, washed 5 times with autoclaved water and laid out under sterile conditions on square plates. Seedlings were grown in the dark at 22°C for up to 25 days or in long-day conditions at 22°C for 5 days at 80 µE on plates containing ½ strength Murashige-Skoog media [34] and 1.5% sucrose solidified with 0.38% gelrite (Duchefa, Harlem, The Netherlands). The media were supplemented with or without PSK-α (NeoMPS, Strasbourg, France) at the concentrations indicated. Epidermal cell lengths were determined directly on hypocotyls using an Olympus microscope BX41 with ten-fold magnification and the image software Cell A (Olympus, Hamburg, Germany). For protoplast experiments, seedlings of *Zea mays* (cv. Garant) were grown in the dark at 26°C for 5 days [22].

### Molecular analysis and GUS staining

P:GUS lines were generated and analyzed as described [10]. β-Glucuronidase activity was determined in shoots of 5-day-old etiolated Arabidopsis seedlings using whole mounts. For cell-type specific analysis of GUS expression, hypocotyls were embedded in Technovit 7100 according to manufacturers' instructions (Heraeus Kulzer). 25 µm thick sections were cut with a Leica RM 2255 microtome and analyzed using a Leica DM LS microscope. Images were taken with a Leica DC 300F camera and Leica IM 500 software (Leica Microsystems).

RT-PCR expression analysis of 5-day-old etiolated seedling organs was performed on total RNA reverse transcribed with an oligo dT primer. The cDNA was amplified with the forward primer 5′-CAAAGACCAGCTCTTCCATCG-3′ and the reverse primer 5′-CTGTGAACGATTCCTGGACCT-3′ for *Actin2*, with the forward primer 5′-GAGCGTTGCAATACAATCAG-3′ and the reverse primer 5′-CAGTACTTACATGCGTCTCGT-3′ for *PSKR1* cDNA, and with the forward primer 5′-GAGGAGACTATCAGCGGGG-3′ and the reverse primer 5′-TCATTGTTGTTGAACAGACTCC-3′ for *PSKR2*. PCR amplifications were performed for 32 cycles for *Actin2* and 38 cycles for the PSK receptors as described [10].

### Effector treatments and protoplast analysis

Protoplasts were isolated from hypocotyls of 5-day-old etiolated Arabidopsis seedlings or from coleoptiles of 5-day-old *Zea mays* seedlings. Protoplasts were isolated by digesting the tissues with cellulase ‘Onozuka’ RS for Arabidopsis protoplasts or with ‘Onozuka’ R-10 for maize protoplasts (Duchefa) and pectolyase (Kikkoman Corporation) for 3 h and 3.5 h, respectively. Net volume changes of protoplasts were analyzed as described [22]. Only vital protoplasts showing strong cytoplasmic streaming were used for the experiments. Stock solutions of PSK-α, unsulfated PSK peptide (usPSK), fusicoccin (FC), ortho-vanadate, cycloheximide, and cholecystokinin octapeptide (CCK8) (Sigma Aldrich) were prepared in washing solution or in washing solution without potassium (1 mM CaCl_2_·2H_2_O, 10 mM MES, pH 6.5, adjusted with Bis-Tris) and diluted to the concentrations indicated. CCK8 was used at 100 nM as described [26].

### pH measurements

Arabidopsis seedlings were grown for 5 days in dim-light conditions (16 h 30 µE light) at 22°C which result in significant hypocotyl growth induction by 10 nM PSK-α (data not shown). *Zea mays* seedlings were grown for 5 days in the dark [22]. Arabidopsis hypocotyls were excised. Maize coleoptile cylinders without 3 mm of the tip and 10 mm long were abraded. pH changes were measured in 350 µl buffer (1 mM CaCl_2_·2H_2_O, 10 mM KCl) containing 60 Arabidopsis hypocotyls per experiment or 8 maize coleoptiles in 1.25 ml buffer using a Beckman pH meter (300 series). PSK-α was diluted in buffer adjusted to the pH of the acid equilibrium and applied at the concentration indicated. The ΔpH was calculated at the times indicated.

### Statistical analysis

Statistical analysis of net volume change of protoplasts was performed with Minitab (Minitab Inc.). For a comparison between treatments, values at t = 35 min for Arabidopsis and at t = 30 min for maize were statistically analyzed. Comparison of means was analyzed for statistical significance with an ANOVA (Tukey test) or a 2-sample *t*-test. Constant variance and normal distribution of data were verified before statistical analysis and the *P* value was set to *P*<0.001 if one of both conditions was not achieved. The *P* value for the Pearson product moment correlation is indicated in the figure legends.

## Supporting Information

Supporting Information S1 Contains Table S1, Figure S1, and Figure S2.
(PDF)
