# Supplementary material for: Phytosulfokine-α Controls Hypocotyl Length and Cell Expansion in Arabidopsis thaliana through Phytosulfokine Receptor 1
Source: PLoS One. 2011 Jun 16;6(6):e21054. doi: 10.1371/journal.pone.0021054 (PMC3116886; doi:10.1371/journal.pone.0021054)

## Supporting Information S1

**Table S1. Cell lengths and cell numbers in hypocotyls of wt, *pskr1-2*, *pskr1-3,* and *pskr2-1*.** Hypocotyl cell lengths of 20 five-day-old etiolated seedlings each were analyzed from wt, *pskr1-2*, *pskr1-3* and *pskr2-1* at each cell position (data set from Figure 1C). ' * ' and ' ° ' indicate significantly different values (*$P$<0.05, °$P$<0.001, 2-sample *t*-test). Hypocotyl lengths (±SE) were calculated by adding average cell lengths from each cell position. Total cell numbers are not statistically different between genotypes ($P$<0.05, Tukey test).

| | Cell length [µm] | | | |
|---|---|---|---|---|
| Cell position | wt | *pskr1-2* | *pskr1-3* | *pskr2-1* |
| 1 | 186.6 | 194.0 | 146.1 | 176.3 |
| 2 | 271.0 | 275.7 | 215.2 | 288.4 |
| 3 | 382.4 | 291.3* | 263.6* | 354.2 |
| 4 | 434.4 | 371.9 | 287.3* | 410.3 |
| 5 | 497.4 | 470.2 | 322.7* | 462.0 |
| 6 | 564.5 | 459.5* | 363.2* | 503.0 |
| 7 | 586.2 | 482.1* | 370.2* | 506.1 |
| 8 | 614.1 | 597.0 | 414.1° | 580.3 |
| 9 | 680.8 | 640.3 | 478.4* | 581.2 |
| 10 | 702.9 | 726.7 | 485.0* | 697.5 |
| 11 | 847.7 | 721.8 | 530.1* | 786.6 |
| 12 | 927.9 | 707.3* | 538.2* | 750.9 |
| 13 | 900.3 | 551.3* | 524.4* | 729.4* |
| 14 | 770.8 | 512.7* | 522.7* | 744.9 |
| 15 | 613.5 | 266.9° | 529.3 | 699.5 |
| 16 | 351.7 | 213.6 | 392.1 | 470.6 |
| 17 | 204.1 | 172.6 | 277.4 | 306.7 |
| 18 | 148.2 | 116.9 | 190.8 | 214.8 |
| 19 | 85.7 | 116.7 | 114.0 | 166.8 |
| 20 | 55.8 | - | - | 151.8 |
| Hypocotyl length [mm] | 9.8 ± 0.2 | 7.9 ± 0.2 | 6.9 ± 0.2 | 9.6 ± 0.1 |
| Total cell number | 19.6 ± 0.3 | 18.8 ± 0.3 | 19.0 ± 0.2 | 20.3 ± 0.3 |

**Figure S1. Time course of hypocotyl elongation of wt, *pskr1-2*, *pskr1-3,* and *pskr2-1* seedlings.** Hypocotyl lengths of 5-, 10-, 15-, 20- and 25-day-old etiolated seedlings were analyzed for wt, *pskr1-2*, *pskr1-3* and *pskr2-1*. Results are averages of hypocotyl lengths (± SE) of a minimum of 36 hypocotyls analyzed per genotype in two independent biological experiments. Error bars are smaller than the symbols used. Hypocotyl lengths of wt and *pskr2-1* are not significant different, whereas hypocotyl lengths of *pskr1-2* and *pskr1-3* seedlings are significant different to wt at each time point ($P<0.001$, 2-sample *t*-test).

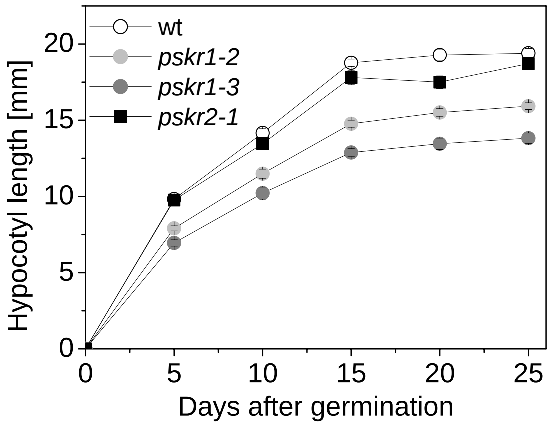

**Figure S2. *t*-test of hypocotyl cell lengths of the allelic lines *pskr1-2* and *pskr1-3*.**

Hypocotyl cell lengths of 5-day-old etiolated seedlings were compared for *pskr1-2* and *pskr1-3* at each cell position (data set from Figure 1C, Table S1). Asterisks indicate significantly different values (*P*<0.05, 2-sample *t*-test).

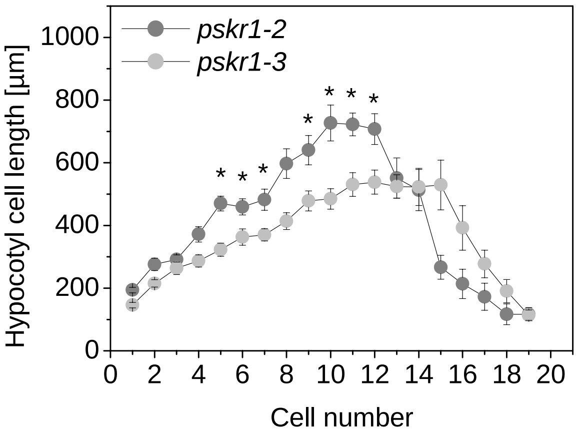

Supplement: Supporting Information S1 — Contains Table S1, Figure S1, and Figure S2. (PDF) [file pone.0021054.s001.pdf]
